# Supplementary material for: Automatic design of gene regulatory mechanisms for spatial pattern formation
Source: NPJ Syst Biol Appl. 2024 Apr 2;10:35. doi: 10.1038/s41540-024-00361-5 (PMC10987498; doi:10.1038/s41540-024-00361-5)
Supplement: Supplementary file 1 — Supplementary Information [file 41540_2024_361_MOESM1_ESM.pdf]

Automatic design of gene regulatory mechanisms for spatial pattern formation

Reza Mousavi and Daniel Lobo

Supplementary Information

This supplement includes additional figures and the system of equations for each of the GRMs presented in the paper.

Contents

1. Supplementary figures 2
2. System of equations for all GRMs 12

1. Supplementary figures

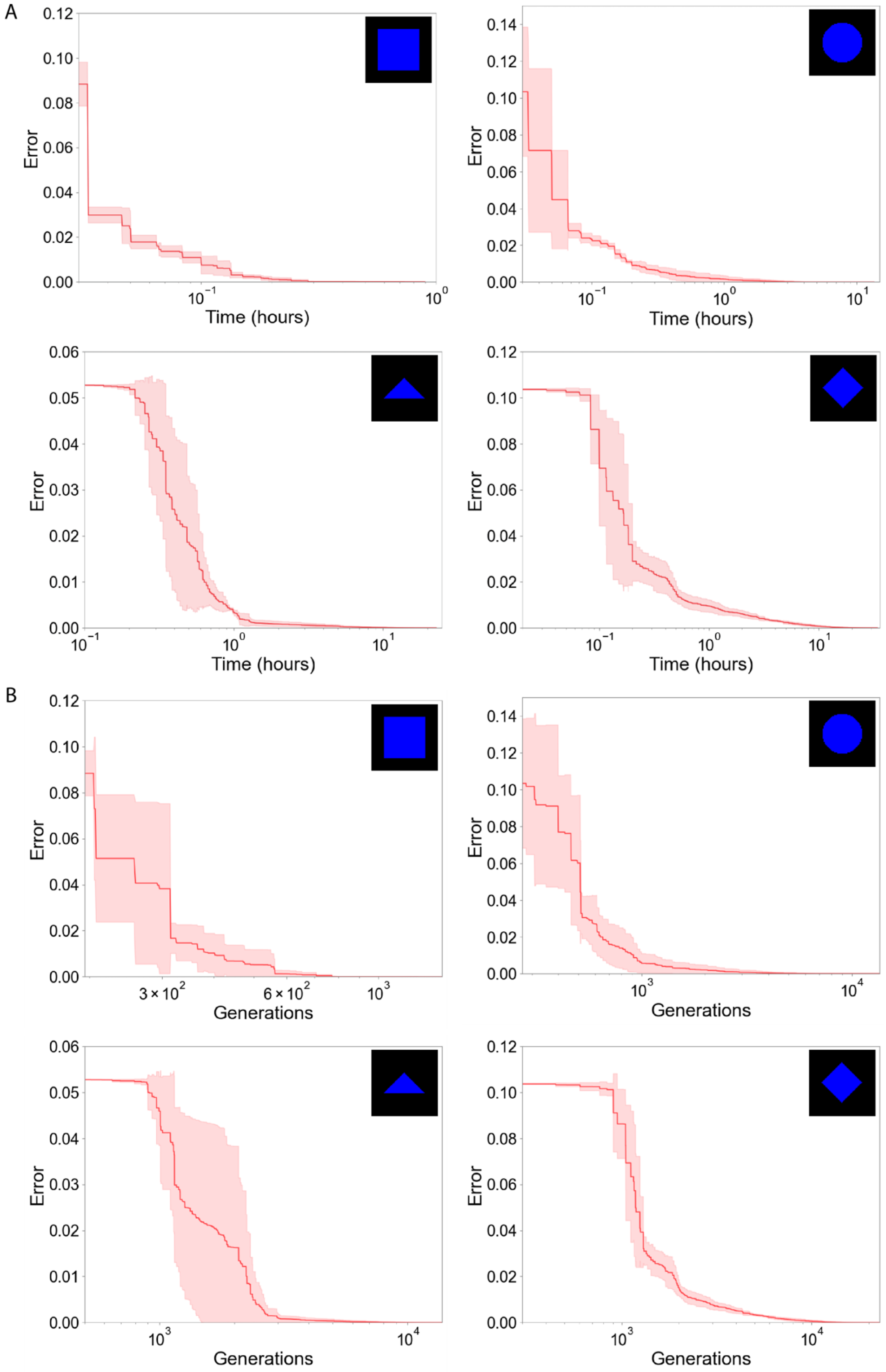

Supplementary Figure 1. Evolutionary dynamics in terms of time (A) and generations (B) for the design of GRMs producing geometric target shapes. Average error of the best GRM across three independent runs of the algorithm, all reaching zero error. Each plot has different y-axis and x-axis ranges for better visualization. The shaded area represents the standard deviation. Error parameters: $k = 5$, $\alpha = 0.1$, $\beta = 0.001$.

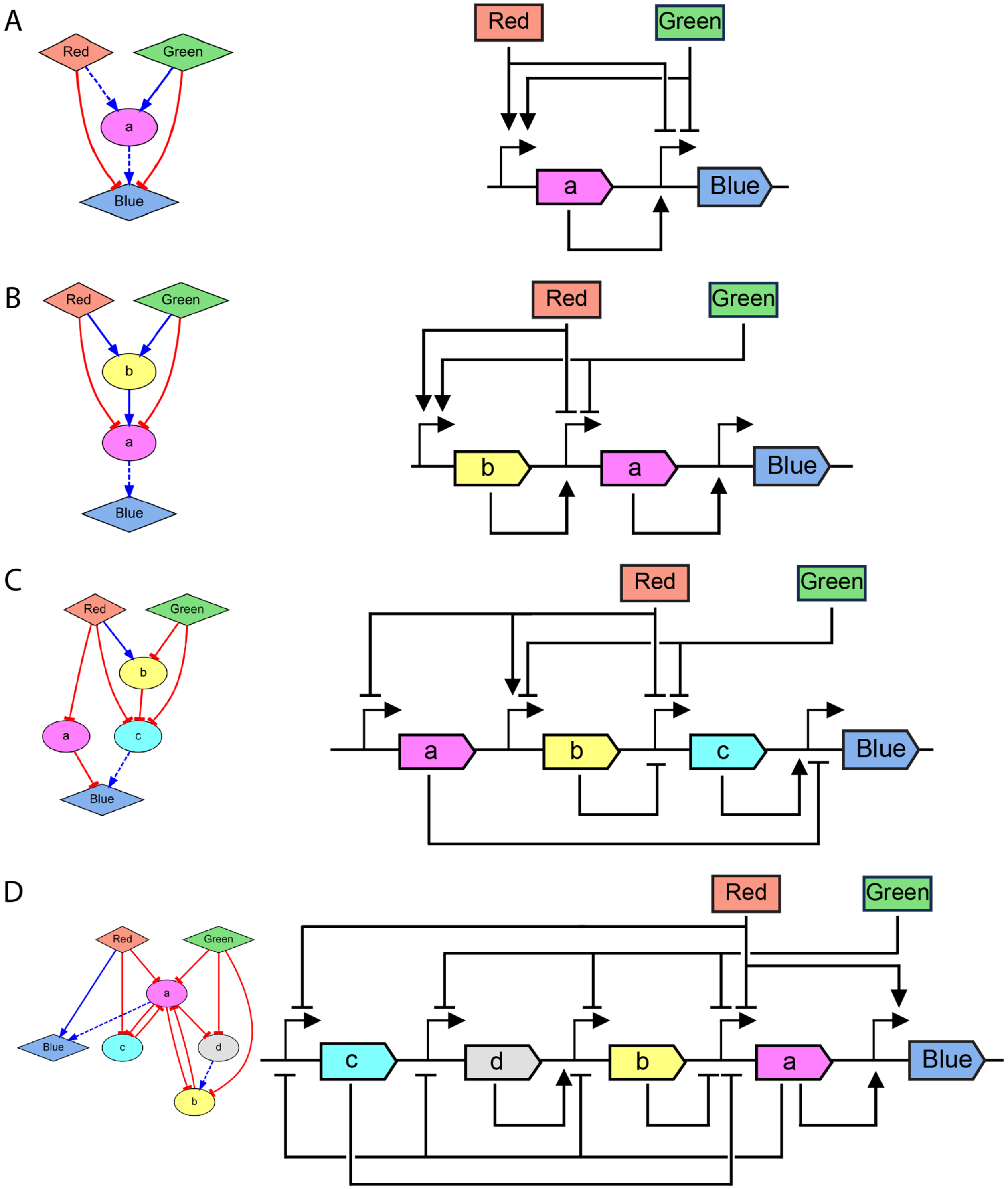

Supplementary Figure 2. Synthetic realizations of GRMs producing geometric target shapes. A-D. GRMs automatically designed (see Fig. 5) and their possible synthetic realizations in SBOL notation.

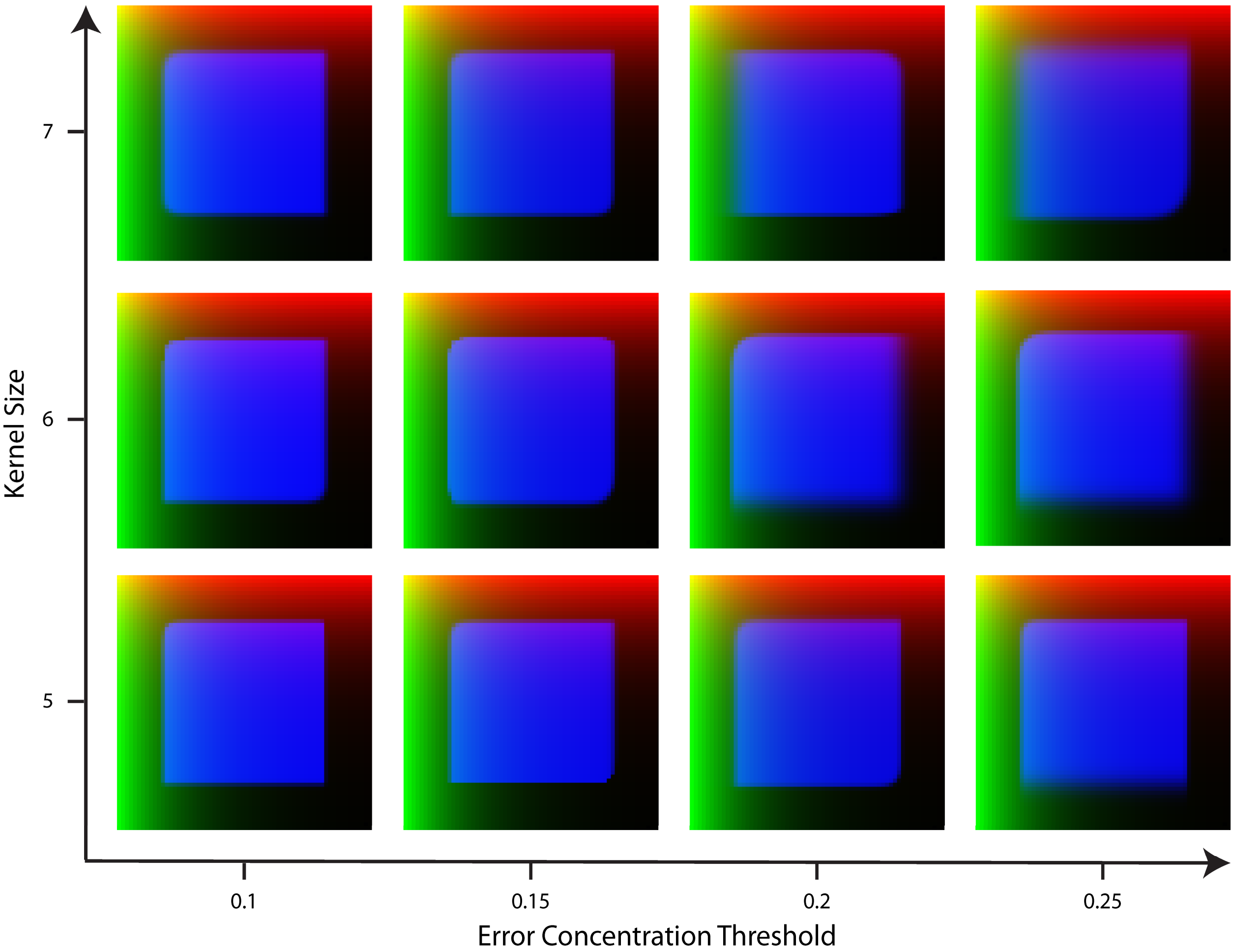

Supplementary Figure 3. Patterns developed by GRMs discovered with the automated methodology for a square target pattern using different values of kernel size and error concentration thresholds.

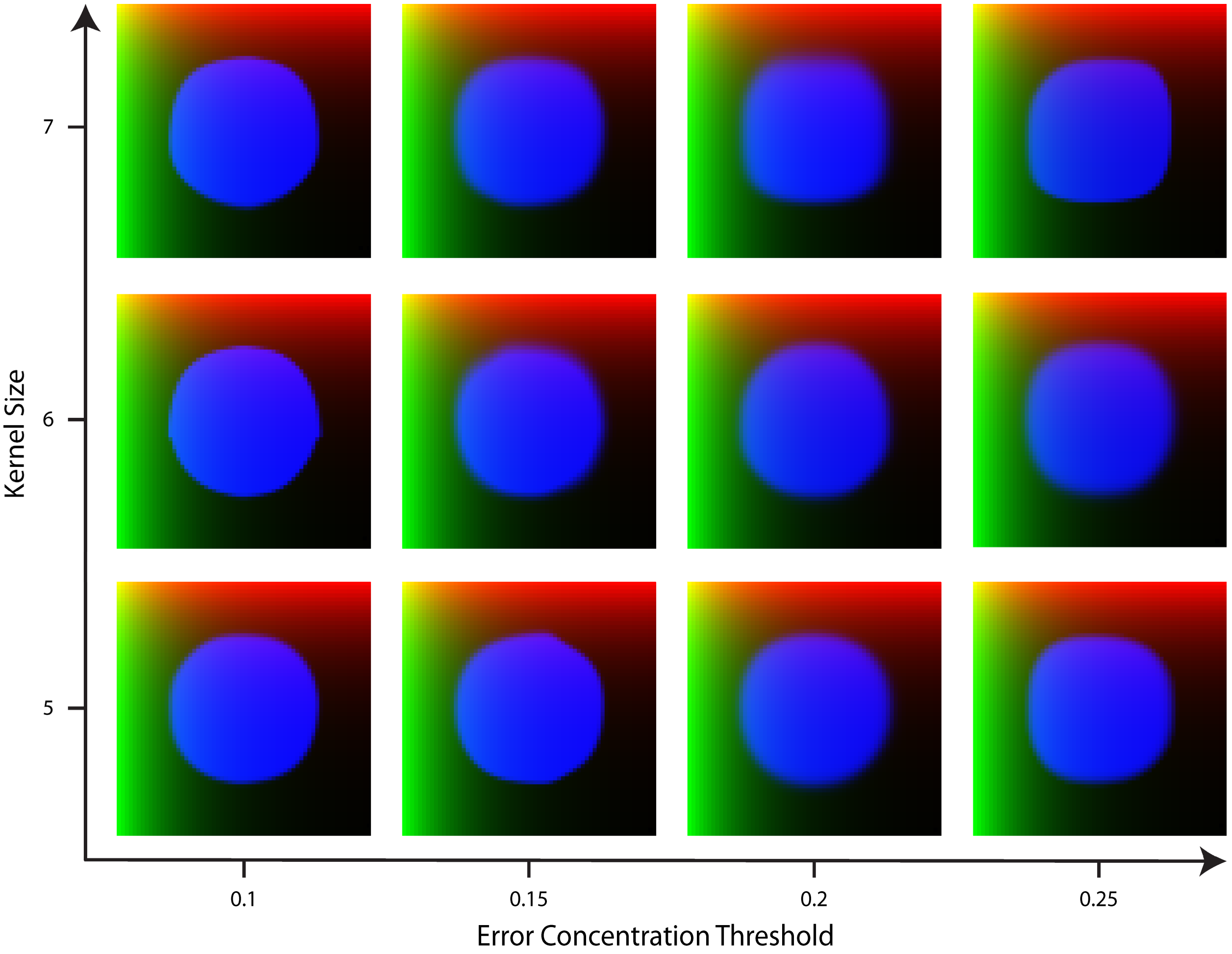

Supplementary Figure 4. Developed patterns by GRMs discovered with the automated methodology for a circle target pattern using different values of kernel size and error concentration thresholds.

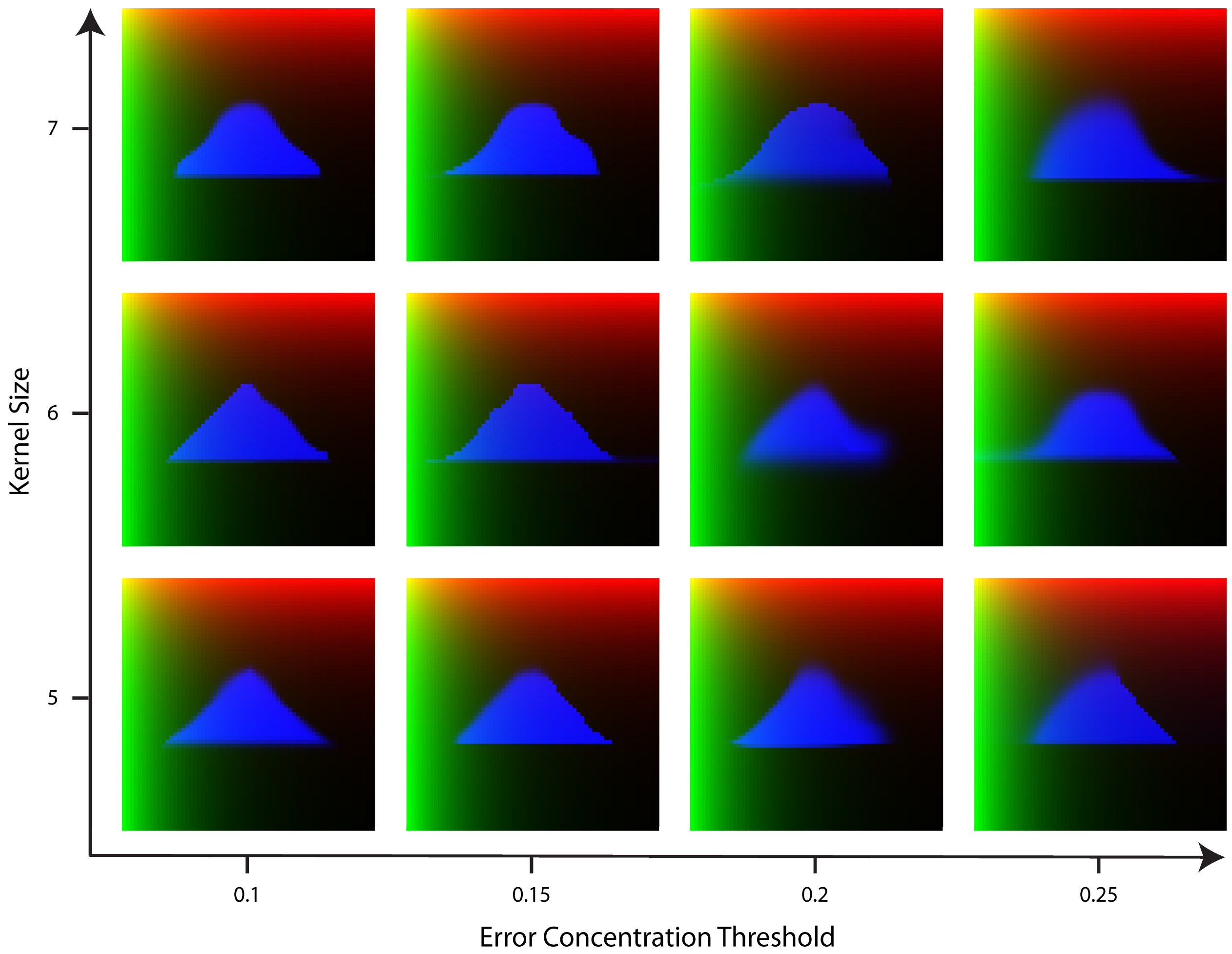

Supplementary Figure 5. Developed patterns by GRMs discovered with the automated methodology for a triangle target pattern using different values of kernel size and error concentration thresholds.

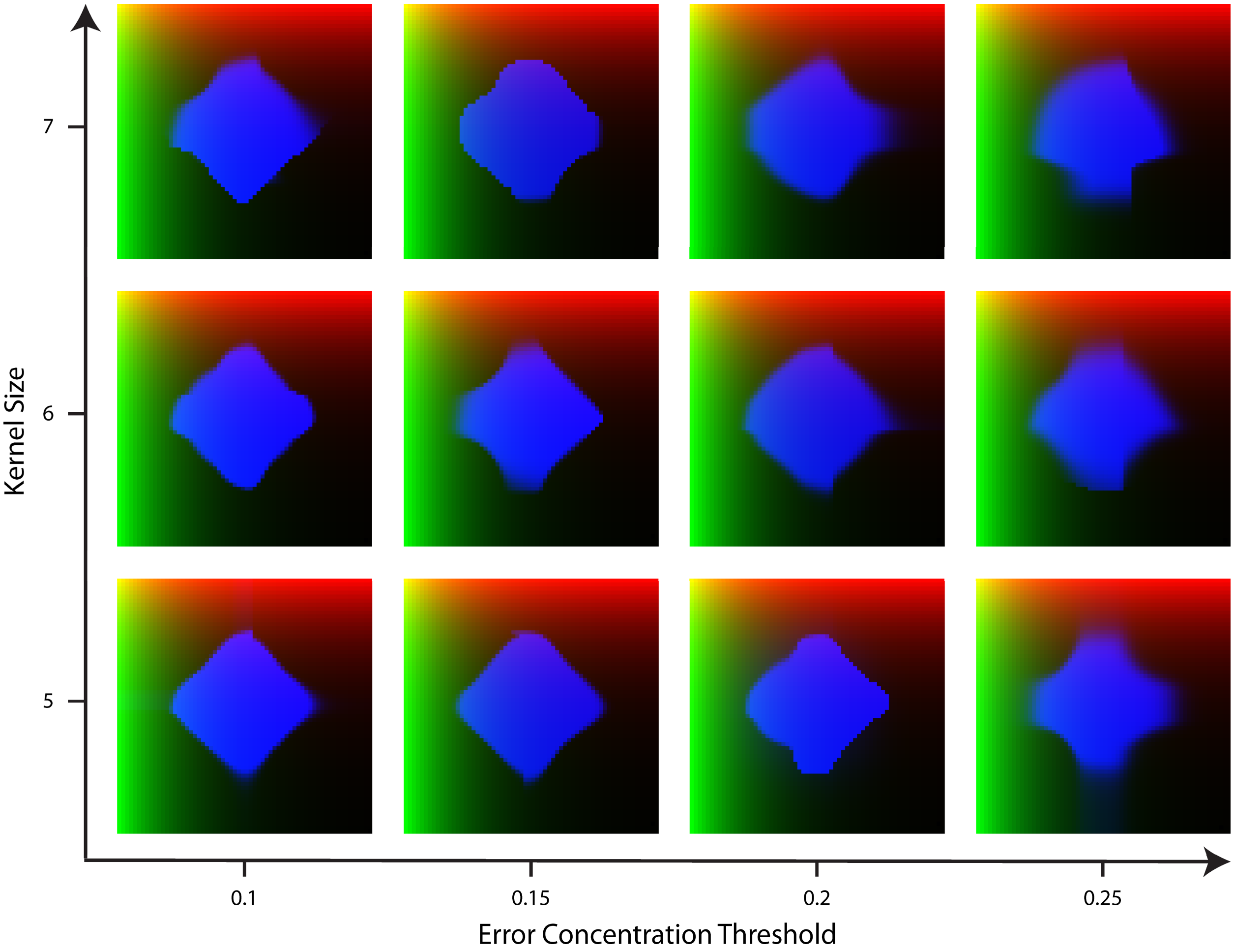

Supplementary Figure 6. Developed patterns by GRMs discovered with the automated methodology for a diamond target pattern using different values of kernel size and error concentration thresholds.

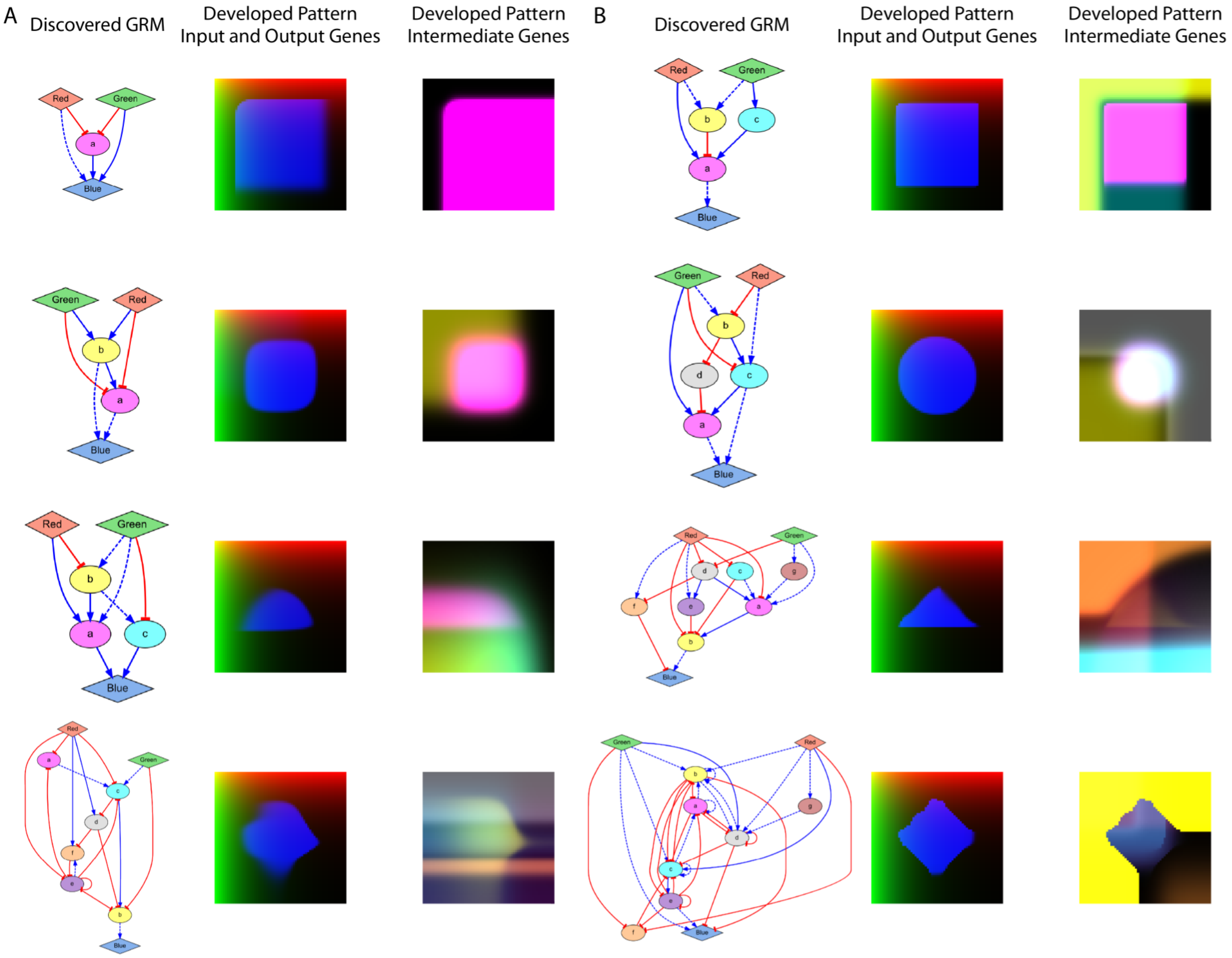

Supplementary Figure 7. Automatically designed GRMs for different geometric target shapes using a limited subset of Hill coefficients. When the method is limited to regulatory interactions using only Hill coefficients $\eta_i \in (1, 2, 4, 8, 10)$, it still can design GRMs resulting in patterns with zero error for all simple (A) and complex (B) geometric target shapes. Expression colors in the developed patterns for the input and output genes and the intermediate genes correspond to the node colors in the GRM network diagrams. Parameters: $k = 7, \alpha = 0.25$ for (A); $k = 5, \alpha = 0.1$ for (B).

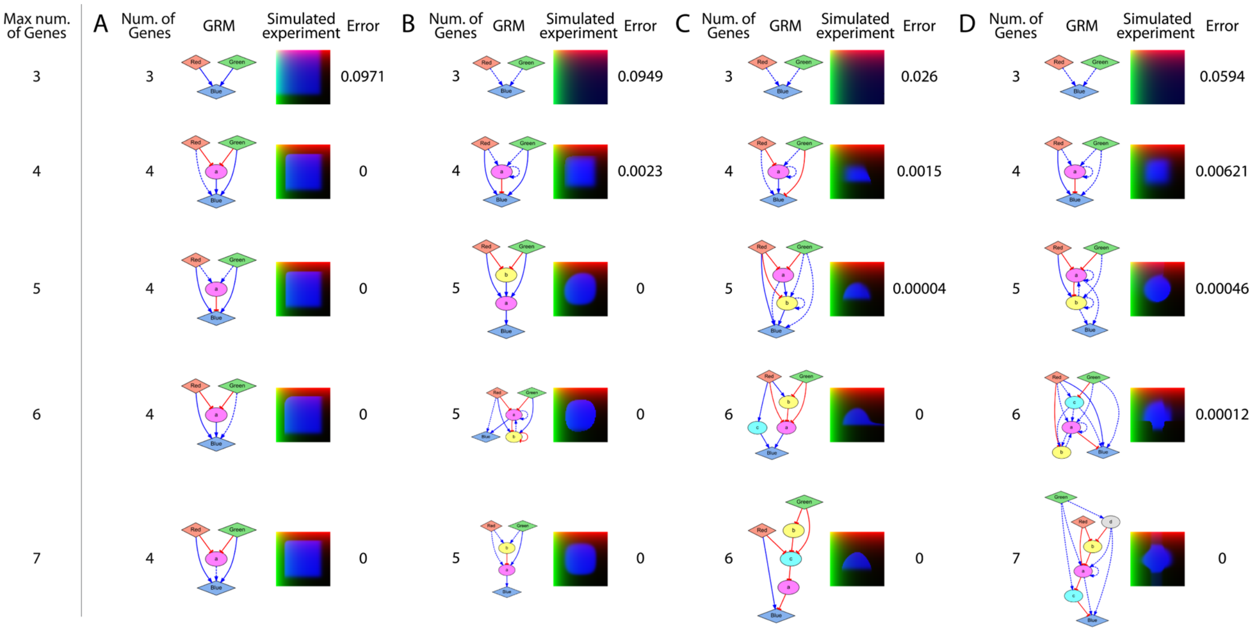

Supplementary Figure 8. Automatically designed GRMs for different geometric target shapes when limiting the maximum number of genes. The error of the resultant pattern anti-correlates with the maximum number of genes allowed in the designed GRMs, yet the designed GRMs do not include more genes than necessary. The target patterns tested include a square (A), circle (B), triangle (C), and diamond (D). Parameters: $k = 7, \alpha = 0.25,\ \beta = 0.001$.

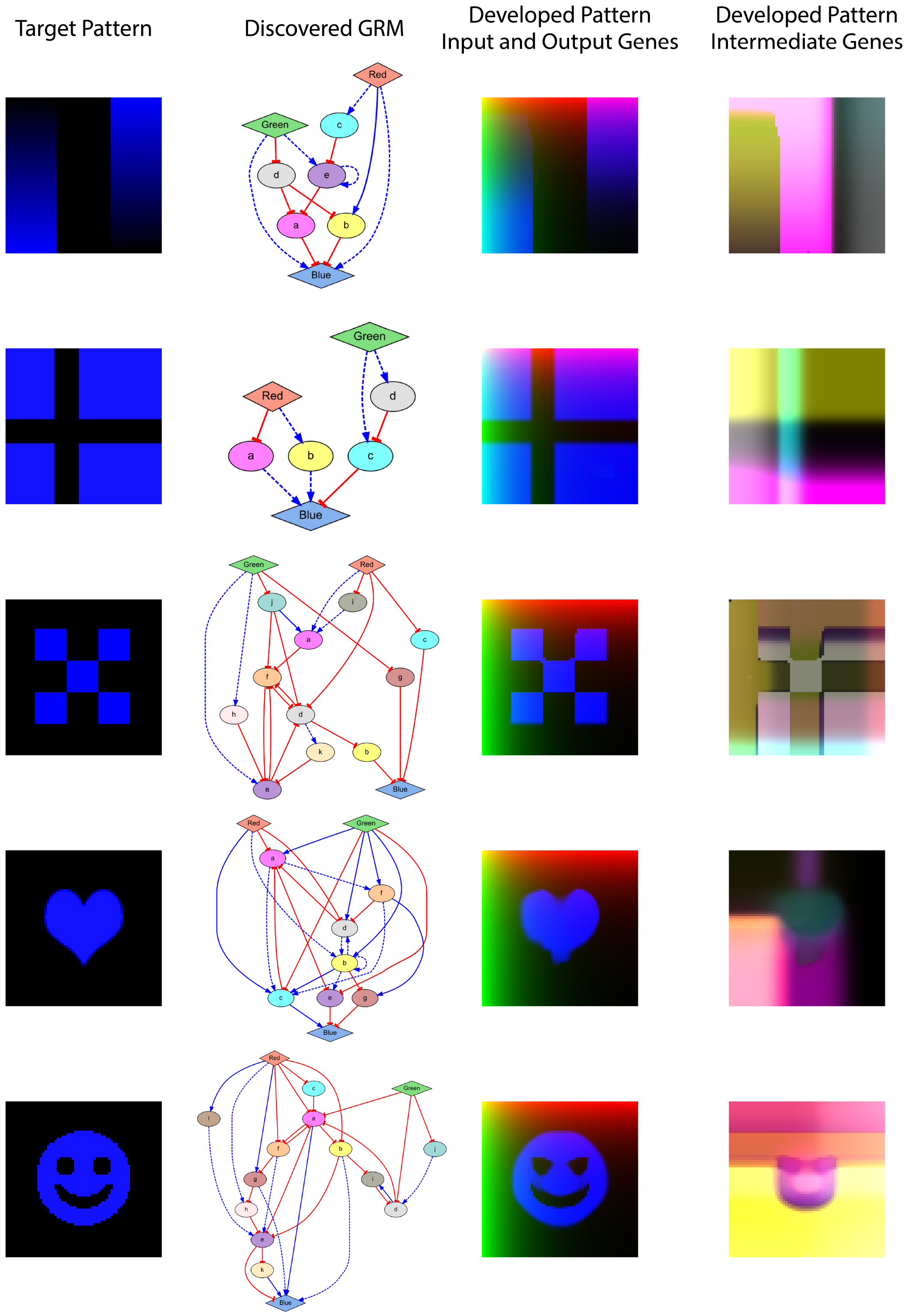

Supplementary Figure 9. Patterns produced by GRMs discovered by the automated methodology applied to arbitrary shapes, including gradients, flag, checkerboard, and symbols. All the resulting GRMs correctly produce a steady-state target pattern (blue) with zero error. Expression colors in the developed patterns for the input and output genes and the intermediate genes correspond to the node colors in the GRM network diagrams. Error parameters: $k = 5$, $\alpha = 0.1$, $\beta = 0.001$.

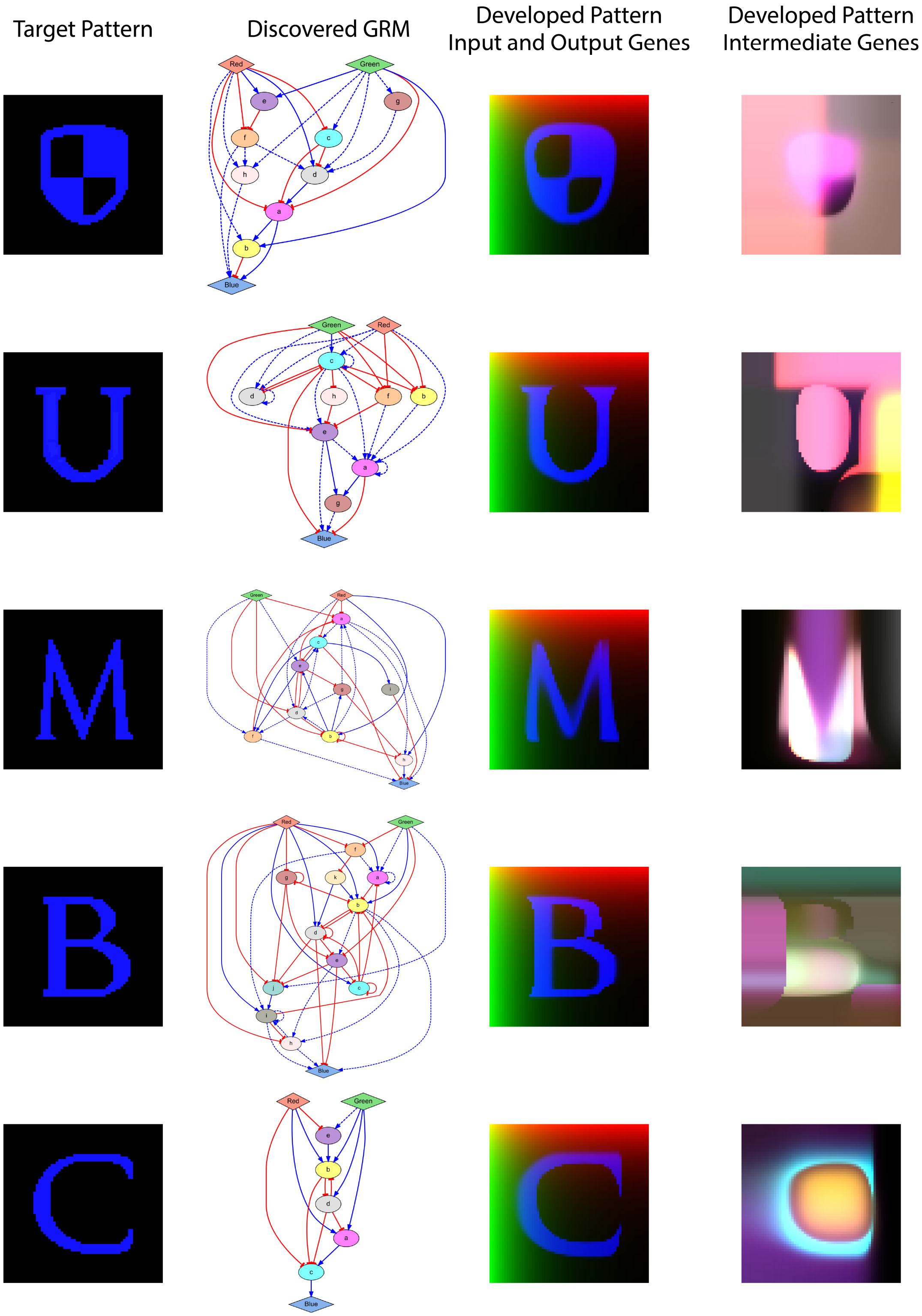

Supplementary Figure 10. Patterns produced by GRMs discovered by the automated methodology applied to arbitrary shapes, including a logo and characters. All the resulting GRMs correctly produce a steady-state target pattern (blue) with zero error. Expression colors in the developed patterns for the input and output genes and the intermediate genes correspond to the node colors in the GRM network diagrams. Error parameters: $k = 5$, $\alpha = 0.1$, $\beta = 0.001$.

2. System of equations for all GRMs

Triangle pattern (Fig. 4B):

$$\frac{\partial Blue}{\partial t} = 0.099 \cdot \frac{90 \cdot e^{6.9} \cdot 62 \cdot d^{9.9}}{(1 + 90 \cdot e^{6.9}) \cdot (1 + 62 \cdot d^{9.9})} - 0.1 \cdot Blue$$

$$\frac{\partial a}{\partial t} = 0.038 \cdot \frac{(3.4 \cdot Green^{4.1} \cdot 28 \cdot c^2 + 3.4 \cdot Green^{4.1} + 28 \cdot c^2)}{(1 + 3.4 \cdot Green^{4.1}) \cdot (1 + 28 \cdot c^2)} - 0.24 \cdot a$$

$$\frac{\partial b}{\partial t} = 0.026 \cdot \frac{\left((5.6 \cdot Red^2 \cdot 2.9 \cdot Green^{2.5} + 5.6 \cdot Red^2 + 2.9 \cdot Green^{2.5}) \cdot 54 \cdot a^{2.8} + (5.6 \cdot Red^2 \cdot 2.9 \cdot Green^{2.5} + 5.6 \cdot Red^2 + 2.9 \cdot Green^{2.5}) + 54 \cdot a^{2.8}\right)}{(1 + 5.6 \cdot Red^2) \cdot (1 + 2.9 \cdot Green^{2.5}) \cdot (1 + 54 \cdot a^{2.8}) \cdot (1 + 17 \cdot d^{2.9}) \cdot (1 + 7.6 \cdot f^{1.5})} - 0.75 \cdot b$$

$$\frac{\partial c}{\partial t} = 0.099 \cdot \frac{1}{(1 + 37 \cdot Green^{3.2}) \cdot (1 + 23 \cdot d^{4.2})} - 0.48 \cdot c$$

$$\frac{\partial d}{\partial t} = 0.053 \cdot \frac{37 \cdot e^{9.6}}{(1 + 37 \cdot e^{9.6}) \cdot (1 + 99 \cdot a^{2.2}) \cdot (1 + 90 \cdot b^{3.2}) \cdot (1 + 62 \cdot c^{1.1})} - 0.43 \cdot d$$

$$\frac{\partial e}{\partial t} = 0.088 \cdot \frac{\left((19 \cdot Red^{9.9} \cdot 42 \cdot b^{6.8} + 19 \cdot Red^{9.9} + 42 \cdot b^{6.8}) \cdot 3.1 \cdot c^{1.6} + (19 \cdot Red^{9.9} \cdot 42 \cdot b^{6.8} + 19 \cdot Red^{9.9} + 42 \cdot b^{6.8}) + 3.1 \cdot c^{1.6}\right)}{(1 + 19 \cdot Red^{9.9}) \cdot (1 + 42 \cdot b^{6.8}) \cdot (1 + 3.1 \cdot c^{1.6})} - 0.93 \cdot e$$

$$\frac{\partial f}{\partial t} = 0.036 \cdot \frac{45 \cdot c^{8.7}}{(1 + 45 \cdot c^{8.7})} - 0.68 \cdot f$$

Approximate square pattern (Fig. 5A):

$$\frac{\partial Blue}{\partial t} = 0.096 \cdot \frac{5.8 \cdot a^{8.4}}{(1 + 5.8 \cdot a^{8.4}) \cdot (1 + 2 \cdot Red^{9.6}) \cdot (1 + 2 \cdot Green^{9.6})} - 0.11 \cdot Blue$$

$$\frac{\partial a}{\partial t} = 0.093 \cdot \frac{40 \cdot Red^5 \cdot 40 \cdot Green^5}{(1 + 40 \cdot Red^5) \cdot (1 + 40 \cdot Green^5)} - 0.16 \cdot a$$

Approximate circle pattern (Fig. 5B):

$$\frac{\partial Blue}{\partial t} = 0.096 \cdot \frac{51 \cdot a^{7.9}}{(1 + 51 \cdot a^{7.9})} - 0.11 \cdot Blue$$

$$\frac{\partial a}{\partial t} = 0.049 \cdot \frac{45 \cdot b^{5.8}}{(1 + 4.6 \cdot Red^4) \cdot (1 + 4.6 \cdot Green^4) \cdot (1 + 45 \cdot b^{5.8})} - 0.18 \cdot a$$

$$\frac{\partial b}{\partial t} = 0.019 \cdot \frac{36 \cdot Red^3 \cdot 36 \cdot Green^3}{(1 + 36 \cdot Red^3) \cdot (1 + 36 \cdot Green^3)} - 0.65 \cdot b$$

Approximate triangle pattern (Fig. 5C):

$$\frac{\partial Blue}{\partial t} = 0.1 \cdot \frac{34 \cdot c^{9.6}}{(1 + 34 \cdot c^{9.6}) \cdot (1 + 94 \cdot a^{9.7})} - 0.11 \cdot Blue$$

$$\frac{\partial a}{\partial t} = 0.073 \cdot \frac{1}{(1 + 36 \cdot Red^9)} - 0.1 \cdot a$$

$$\frac{\partial b}{\partial t} = 0.078 \cdot \frac{17 \cdot Red^{4.4}}{(1 + 17 \cdot Red^{4.4}) \cdot (1 + 45 \cdot Green^{2.5})} - 0.53 \cdot b$$

$$\frac{\partial c}{\partial t} = 0.079 \cdot \frac{1}{(1 + 12 \cdot Red^{1.2}) \cdot (1 + 3.5 \cdot Green^{2.2}) \cdot (1 + 49 \cdot b^{1.9})} - 0.71 \cdot c$$

Approximate diamond pattern (Fig. 5D):

$$\frac{\partial Blue}{\partial t} = 0.096 \cdot \frac{44 \cdot a^{9.3} \cdot 33 \cdot Red^{9.4}}{(1 + 44 \cdot a^{9.3}) \cdot (1 + 33 \cdot Red^{9.4})} - 0.1 \cdot Blue$$

$$\frac{\partial a}{\partial t} = 0.013 \cdot \frac{1}{(1 + 3.9 \cdot Red^{2.1}) \cdot (1 + 3.9 \cdot Green^{2.1}) \cdot (1 + 25 \cdot b^{1.6}) \cdot (1 + 51 \cdot c^{4})} - 0.17 \cdot a$$

$$\frac{\partial b}{\partial t} = 0.072 \cdot \frac{28 \cdot d^{7.4}}{(1 + 28 \cdot d^{7.4}) \cdot (1 + 52 \cdot Green^{1.3}) \cdot (1 + 19 \cdot a^{4.3})} - 0.39 \cdot b$$

$$\frac{\partial c}{\partial t} = 0.009 \cdot \frac{1}{(1 + 15 \cdot Red^{9.7}) \cdot (1 + 19 \cdot a^{4.3})} - 0.42 \cdot c$$

$$\frac{\partial d}{\partial t} = 0.07 \cdot \frac{1}{(1 + 14 \cdot Green^{10}) \cdot (1 + 27 \cdot a^{1.7})} - 0.9 \cdot d$$

Precise square pattern (Fig. 6A):

$$\frac{\partial Blue}{\partial t} = 0.099 \cdot \frac{1}{(1 + 52 \cdot a^{9.5}) \cdot (1 + 64 \cdot b^{8.1})} - 0.11 \cdot Blue$$

$$\frac{\partial a}{\partial t} = 0.063 \cdot \frac{\left((1.9 \cdot Red^{9.5} \cdot 1.9 \cdot Green^{9.4} + 1.9 \cdot Red^{9.5} + 1.9 \cdot Green^{9.4}) \cdot 30 \cdot c^{5.8} + (1.9 \cdot Red^{9.5} \cdot 1.9 \cdot Green^{9.4} + 1.9 \cdot Red^{9.5} + 1.9 \cdot Green^{9.4}) + 30 \cdot c^{5.8}\right)}{(1 + 1.9 \cdot Red^{9.5}) \cdot (1 + 1.9 \cdot Green^{9.4}) \cdot (1 + 30 \cdot c^{5.8})} - 0.51 \cdot a$$

$$\frac{\partial b}{\partial t} = 0.032 \cdot \frac{1}{(1 + 47 \cdot Red^{9.5})} - 0.63 \cdot b$$

$$\frac{\partial c}{\partial t} = 0.04 \cdot \frac{1}{(1 + 59 \cdot Green^{7.6})} - 0.13 \cdot c$$

Precise circle pattern (Fig. 6B):

$$\frac{\partial Blue}{\partial t} = 0.1 \cdot \frac{88 \cdot b^{7.1}}{(1 + 88 \cdot b^{7.1})} - 0.1 \cdot Blue$$

$$\frac{\partial a}{\partial t} = 0.059 \cdot \frac{29 \cdot Green^{2.5} \cdot 29 \cdot Red^{2.5}}{(1 + 29 \cdot Green^{2.5}) \cdot (1 + 29 \cdot Red^{2.5})} - 0.76 \cdot a$$

$$\frac{\partial b}{\partial t} = 0.077 \cdot \frac{16 \cdot a^{4.1}}{(1 + 5.1 \cdot Red^{4.9}) \cdot (1 + 5.1 \cdot Green^{4.9}) \cdot (1 + 16 \cdot a^{4.1}) \cdot (1 + 96 \cdot c^{4.9})} - 0.14 \cdot b$$

$$\frac{\partial c}{\partial t} = 0.0093 \cdot \frac{(9.6 \cdot Red^{4.5} \cdot 9.5 \cdot Green^{4.9} + 9.6 \cdot Red^{4.5} + 9.5 \cdot Green^{4.9})}{(1 + 9.6 \cdot Red^{4.5}) \cdot (1 + 9.5 \cdot Green^{4.9}) \cdot (1 + 14 \cdot a^{6.2})} - 0.99 \cdot c$$

Precise triangle pattern (Fig. 6C):

$$\frac{\partial Blue}{\partial t} = 0.1 \cdot \frac{1}{(1 + 80 \cdot a^{9.4}) \cdot (1 + 17 \cdot b^{10}) \cdot (1 + 1\mathrm{e}+02 \cdot c^{10})} - 0.1 \cdot Blue$$

$$\frac{\partial a}{\partial t} = 0.051 \cdot \frac{65 \cdot e^{8.4}}{(1 + 65 \cdot e^{8.4}) \cdot (1 + 69 \cdot Green^{2.4}) \cdot (1 + 95 \cdot b^{1})} - 0.15 \cdot a$$

$$\frac{\partial b}{\partial t} = 0.044 \cdot \frac{1}{(1 + 28 \cdot Red^{3.6}) \cdot (1 + 40 \cdot a^{9.9}) \cdot (1 + 38 \cdot c^{9.9})} - 0.17 \cdot b$$

$$\frac{\partial c}{\partial t} = 0.058 \cdot \frac{\left(\begin{pmatrix}(1.7 \cdot Red^{3.2} \cdot 1.7 \cdot Green^{3.2} + 1.7 \cdot Red^{3.2} + 1.7 \cdot Green^{3.2}) \cdot 17 \cdot a^{1.7} + \\ (1.7 \cdot Red^{3.2} \cdot 1.7 \cdot Green^{3.2} + 1.7 \cdot Red^{3.2} + 1.7 \cdot Green^{3.2}) + 17 \cdot a^{1.7}\end{pmatrix} \cdot 1.7 \cdot d^{3.2} + \begin{pmatrix}(1.7 \cdot Red^{3.2} \cdot 1.7 \cdot Green^{3.2} + 1.7 \cdot Red^{3.2} + 1.7 \cdot Green^{3.2}) \cdot 17 \cdot a^{1.7} + \\ (1.7 \cdot Red^{3.2} \cdot 1.7 \cdot Green^{3.2} + 1.7 \cdot Red^{3.2} + 1.7 \cdot Green^{3.2}) + 17 \cdot a^{1.7}\end{pmatrix} + 1.7 \cdot d^{3.2}\right)}{(1 + 1.7 \cdot Red^{3.2}) \cdot (1 + 1.7 \cdot Green^{3.2}) \cdot (1 + 17 \cdot a^{1.7}) \cdot (1 + 1.7 \cdot d^{3.2}) \cdot (1 + 1\mathrm{e}+02 \cdot b^{1.4})} - 0.2 \cdot c$$

$$\frac{\partial d}{\partial t} = 0.073 \cdot \frac{1}{(1 + 1\mathrm{e}+02 \cdot b^{3.1}) \cdot (1 + 1\mathrm{e}+02 \cdot e^{1})} - 0.53 \cdot d$$

$$\frac{\partial e}{\partial t} = 0.046 \cdot \frac{1}{(1 + 16 \cdot Green^{7.1})} - 0.16 \cdot e$$

Precise diamond pattern (Fig. 6D):

$$\frac{\partial Blue}{\partial t} = 0.1 \cdot \frac{(87 \cdot a^{10} \cdot 99 \cdot f^{6.3} + 87 \cdot a^{10} + 99 \cdot f^{6.3})}{(1 + 87 \cdot a^{10}) \cdot (1 + 99 \cdot f^{6.3})} - 0.1 \cdot Blue$$

$$\frac{\partial a}{\partial t} = 0.1 \cdot \frac{1}{(1 + 7.1 \cdot Red^{4.1}) \cdot (1 + 7.1 \cdot Green^{4.1}) \cdot (1 + 98 \cdot b^{2.6}) \cdot (1 + 59 \cdot d^{2.9}) \cdot (1 + 60 \cdot e^{2.4})} - 0.12 \cdot a$$

$$\frac{\partial b}{\partial t} = 0.067 \cdot \frac{1}{(1 + 25 \cdot Red^{2.8}) \cdot (1 + 13 \cdot b^{2.4}) \cdot (1 + 13 \cdot c^{1.5})} - 0.88 \cdot b$$

$$\frac{\partial c}{\partial t} = 0.057 \cdot \frac{1}{(1 + 98 \cdot d^{1.9}) \cdot (1 + 99 \cdot e^{2.8}) \cdot (1 + 98 \cdot g^{7.7})} - 0.48 \cdot c$$

$$\frac{\partial d}{\partial t} = 0.038 \cdot \frac{1}{(1 + 25 \cdot Green^{2.5}) \cdot (1 + 13 \cdot c^{7.9}) \cdot (1 + 11 \cdot d^{5.7})} - 0.38 \cdot d$$

$$\frac{\partial e}{\partial t} = 0.0043 \cdot \frac{(6 \cdot Green^{6.3} \cdot 54 \cdot d^{1.3} + 6 \cdot Green^{6.3} + 54 \cdot d^{1.3}) \cdot 6 \cdot Red^{9.3}}{(1 + 6 \cdot Green^{6.3}) \cdot (1 + 54 \cdot d^{1.3}) \cdot (1 + 6 \cdot Red^{9.3})} - 0.21 \cdot e$$

$$\frac{\partial f}{\partial t} = 0.008 \cdot \frac{(57 \cdot a^{5.4} \cdot 6.1 \cdot c^{6.8} + 57 \cdot a^{5.4} + 6.1 \cdot c^{6.8})}{(1 + 57 \cdot a^{5.4}) \cdot (1 + 6.1 \cdot c^{6.8})} - 0.12 \cdot f$$

$$\frac{\partial g}{\partial t} = 0.092 \cdot \frac{12 \cdot Green^{7.9}}{(1 + 16 \cdot Red^{8.6}) \cdot (1 + 12 \cdot Green^{7.9})} - 0.46 \cdot g$$

Gradients pattern (Fig. 8A):

$$\frac{\partial Blue}{\partial t} = 0.095 \cdot \frac{(12 \cdot Red^{1.7} \cdot 5.9 \cdot Green^{9.8} + 12 \cdot Red^{1.7} + 5.9 \cdot Green^{9.8})}{(1 + 12 \cdot Red^{1.7}) \cdot (1 + 5.9 \cdot Green^{9.8}) \cdot (1 + 18 \cdot a^{9.4}) \cdot (1 + 10 \cdot b^{7.5})} - 0.1 \cdot Blue$$

$$\frac{\partial a}{\partial t} = 0.092 \cdot \frac{1}{(1 + 91 \cdot d^{5}) \cdot (1 + 75 \cdot e^{6.4})} - 0.13 \cdot a$$

$$\frac{\partial b}{\partial t} = 0.076 \cdot \frac{39 \cdot Red^{1}}{(1 + 39 \cdot Red^{1}) \cdot (1 + 98 \cdot d^{2.2})} - 0.63 \cdot b$$

$$\frac{\partial c}{\partial t} = 0.025 \cdot \frac{5 \cdot Red^{1.4}}{(1 + 5 \cdot Red^{1.4})} - 0.96 \cdot c$$

$$\frac{\partial d}{\partial t} = 0.087 \cdot \frac{1}{(1 + 31 \cdot Green^{3.7})} - 0.98 \cdot d$$

$$\frac{\partial e}{\partial t} = 0.048 \cdot \frac{(2.6 \cdot Green^{4.1} \cdot 95 \cdot e^{5} + 2.6 \cdot Green^{4.1} + 95 \cdot e^{5})}{(1 + 2.6 \cdot Green^{4.1}) \cdot (1 + 95 \cdot e^{5}) \cdot (1 + 49 \cdot c^{7.6})} - 0.99 \cdot e$$

Flag pattern (Fig. 8A):

$$\frac{\partial Blue}{\partial t} = 0.098 \cdot \frac{(90 \cdot a^{9.9} \cdot 18 \cdot b^{8.1} + 90 \cdot a^{9.9} + 18 \cdot b^{8.1})}{(1 + 90 \cdot a^{9.9}) \cdot (1 + 18 \cdot b^{8.1}) \cdot (1 + 16 \cdot c^{9.7})} - 0.1 \cdot Blue$$

$$\frac{\partial a}{\partial t} = 0.076 \cdot \frac{1}{(1 + 48 \cdot Red^{3.1})} - 0.22 \cdot a$$

$$\frac{\partial b}{\partial t} = 0.041 \cdot \frac{8.1 \cdot Red^{9.7}}{(1 + 8.1 \cdot Red^{9.7})} - 0.36 \cdot b$$

$$\frac{\partial c}{\partial t} = 0.046 \cdot \frac{8.1 \cdot Green^{9.9}}{(1 + 8.1 \cdot Green^{9.9}) \cdot (1 + 16 \cdot d^{9.7})} - 0.33 \cdot c$$

$$\frac{\partial d}{\partial t} = 0.061 \cdot \frac{3.5 \cdot Green^{3.1}}{(1 + 3.5 \cdot Green^{3.1})} - 0.34 \cdot d$$

Checkerboard pattern (Fig. 8A):

$$\frac{\partial Blue}{\partial t} = 0.1 \cdot \frac{1}{(1 + 74 \cdot b^{7}) \cdot (1 + 74 \cdot c^{4.9}) \cdot (1 + 73 \cdot g^{6})} - 0.1 \cdot Blue$$

$$\frac{\partial a}{\partial t} = 0.053 \cdot \frac{(3.4 \cdot Red^{5.1} \cdot 80 \cdot i^{4.9} + 3.4 \cdot Red^{5.1} + 80 \cdot i^{4.9}) \cdot 54 \cdot j^{9.3}}{(1 + 3.4 \cdot Red^{5.1}) \cdot (1 + 80 \cdot i^{4.9}) \cdot (1 + 54 \cdot j^{9.3})} - 0.25 \cdot a$$

$$\frac{\partial b}{\partial t} = 0.084 \cdot \frac{1}{(1 + 76 \cdot d^{6.6})} - 0.21 \cdot b$$

$$\frac{\partial c}{\partial t} = 0.089 \cdot \frac{1}{(1 + 67 \cdot Red^{7.3})} - 0.12 \cdot c$$

$$\frac{\partial d}{\partial t} = 0.09 \cdot \frac{1}{(1 + 3.2 \cdot Red^{9.9}) \cdot (1 + 89 \cdot e^{9.1}) \cdot (1 + 13 \cdot f^{8.3}) \cdot (1 + 4 \cdot j^{3.6})} - 0.17 \cdot d$$

$$\frac{\partial e}{\partial t} = 0.054 \cdot \frac{12 \cdot Green^{5.4}}{(1 + 12 \cdot Green^{5.4}) \cdot (1 + 22 \cdot f^{8.7}) \cdot (1 + 94 \cdot h^{8.5}) \cdot (1 + 93 \cdot k^{8.4})} - 0.95 \cdot e$$

$$\frac{\partial f}{\partial t} = 0.095 \cdot \frac{1}{(1 + 67 \cdot a^{8.3}) \cdot (1 + 4 \cdot d^{4.1}) \cdot (1 + 87 \cdot e^{9.5}) \cdot (1 + 4 \cdot j^{3.7})} - 0.73 \cdot f$$

$$\frac{\partial g}{\partial t} = 0.08 \cdot \frac{1}{(1 + 59 \cdot Green^{9})} - 0.15 \cdot g$$

$$\frac{\partial h}{\partial t} = 0.086 \cdot \frac{2.5 \cdot Green^{3.6}}{(1 + 2.5 \cdot Green^{3.6})} - 0.29 \cdot h$$

$$\frac{\partial i}{\partial t} = 0.078 \cdot \frac{1}{(1 + 51 \cdot Red^{2.8})} - 0.36 \cdot i$$

$$\frac{\partial j}{\partial t} = 0.097 \cdot \frac{1}{(1 + 2.8 \cdot Green^{9.2})} - 0.75 \cdot j$$

$$\frac{\partial k}{\partial t} = 0.093 \cdot \frac{3 \cdot d^{8.2}}{(1 + 3 \cdot d^{8.2})} - 0.32 \cdot k$$

Heart pattern (Fig. 8A):

$$\frac{\partial Blue}{\partial t} = 0.1 \cdot \frac{18 \cdot c^{10}}{(1 + 18 \cdot c^{10}) \cdot (1 + 23 \cdot e^{7.4}) \cdot (1 + 9 \cdot g^{2.6})} - 0.1 \cdot Blue$$

$$\frac{\partial a}{\partial t} = 0.084 \cdot \frac{29 \cdot Green^{5.2}}{(1 + 9 \cdot Red^{2.6}) \cdot (1 + 29 \cdot Green^{5.2}) \cdot (1 + 6.6 \cdot c^{9.5}) \cdot (1 + 63 \cdot d^{1.5})} - 0.23 \cdot a$$

$$\frac{\partial b}{\partial t} = 0.031 \cdot \frac{\left((13 \cdot Red^{1.8} \cdot 19 \cdot b^{9.9} + 13 \cdot Red^{1.8} + 19 \cdot b^{9.9}) \cdot 64 \cdot d^{6.4} + (13 \cdot Red^{1.8} \cdot 19 \cdot b^{9.9} + 13 \cdot Red^{1.8} + 19 \cdot b^{9.9}) + 64 \cdot d^{6.4}\right) \cdot 23 \cdot Green^{2.8}}{(1 + 13 \cdot Red^{1.8}) \cdot (1 + 19 \cdot b^{9.9}) \cdot (1 + 64 \cdot d^{6.4}) \cdot (1 + 23 \cdot Green^{2.8})} - 0.46 \cdot b$$

$$\frac{\partial c}{\partial t} = 0.055 \cdot \frac{(36 \cdot a^{3.3} \cdot 19 \cdot f^{9.9} + 36 \cdot a^{3.3} + 19 \cdot f^{9.9}) \cdot 18 \cdot Red^{2.5} \cdot 49 \cdot b^{3.1}}{(1 + 36 \cdot a^{3.3}) \cdot (1 + 19 \cdot f^{9.9}) \cdot (1 + 18 \cdot Red^{2.5}) \cdot (1 + 4.5 \cdot Green^{3.2}) \cdot (1 + 49 \cdot b^{3.1})} - 0.2 \cdot c$$

$$\frac{\partial d}{\partial t} = 0.027 \cdot \frac{79 \cdot b^{7.9} \cdot 18 \cdot Green^{8.3}}{(1 + 79 \cdot b^{7.9}) \cdot (1 + 23 \cdot Red^{1.5}) \cdot (1 + 18 \cdot Green^{8.3}) \cdot (1 + 85 \cdot f^{3.9})} - 0.95 \cdot d$$

$$\frac{\partial e}{\partial t} = 0.083 \cdot \frac{18 \cdot b^{10}}{(1 + 18 \cdot b^{10}) \cdot (1 + 7.4 \cdot Green^{6.9}) \cdot (1 + 55 \cdot a^{3.7})} - 0.13 \cdot e$$

$$\frac{\partial f}{\partial t} = 0.081 \cdot \frac{7.9 \cdot a^{10} \cdot 4.1 \cdot Green^{7.5}}{(1 + 7.9 \cdot a^{10}) \cdot (1 + 4.1 \cdot Green^{7.5})} - 0.11 \cdot f$$

$$\frac{\partial g}{\partial t} = 0.088 \cdot \frac{18 \cdot f^{2.4}}{(1 + 35 \cdot b^{9.8}) \cdot (1 + 18 \cdot f^{2.4})} - 0.35 \cdot g$$

Smiley face pattern (Fig. 8A):

$$\frac{\partial Blue}{\partial t} = 0.1 \cdot \frac{(49 \cdot b^{9.5} \cdot 99 \cdot g^{6.5} + 49 \cdot b^{9.5} + 99 \cdot g^{6.5}) \cdot 86 \cdot a^{10} \cdot 53 \cdot k^{8.5}}{(1 + 49 \cdot b^{9.5}) \cdot (1 + 99 \cdot g^{6.5}) \cdot (1 + 86 \cdot a^{10}) \cdot (1 + 5.2 \cdot e^{9.9}) \cdot (1 + 53 \cdot k^{8.5})} - 0.1 \cdot Blue$$

$$\frac{\partial a}{\partial t} = 0.066 \cdot \frac{1}{(1 + 5.2 \cdot Red^{3.6}) \cdot (1 + 5.2 \cdot Green^{3.9}) \cdot (1 + 84 \cdot c^{2.3}) \cdot (1 + 59 \cdot d^{2.9}) \cdot (1 + 5.2 \cdot f^{3.1})} - 0.22 \cdot a$$

$$\frac{\partial b}{\partial t} = 0.098 \cdot \frac{1}{(1 + 7.3 \cdot Red^{9.5}) \cdot (1 + 23 \cdot a^{6.2})} - 0.18 \cdot b$$

$$\frac{\partial c}{\partial t} = 0.044 \cdot \frac{1}{(1 + 41 \cdot Red^{2.4})} - 0.47 \cdot c$$

$$\frac{\partial d}{\partial t} = 0.062 \cdot \frac{71 \cdot j^{5.7}}{(1 + 71 \cdot j^{5.7}) \cdot (1 + 70 \cdot Green^{1.7}) \cdot (1 + 6.1 \cdot i^{5.4})} - 0.38 \cdot d$$

$$\frac{\partial e}{\partial t} = 0.094 \cdot \frac{(53 \cdot f^{9.5} \cdot 69 \cdot l^{9.2} + 53 \cdot f^{9.5} + 69 \cdot l^{9.2})}{(1 + 53 \cdot f^{9.5}) \cdot (1 + 69 \cdot l^{9.2}) \cdot (1 + 5.2 \cdot a^{9.3}) \cdot (1 + 81 \cdot b^{1.7}) \cdot (1 + 5.2 \cdot h^{4.5})} - 0.31 \cdot e$$

$$\frac{\partial f}{\partial t} = 0.077 \cdot \frac{1}{(1 + 6.1 \cdot Red^{5.4}) \cdot (1 + 6.1 \cdot a^{3.1})} - 0.98 \cdot f$$

$$\frac{\partial g}{\partial t} = 0.076 \cdot \frac{7.3 \cdot Red^{4.4}}{(1 + 7.3 \cdot Red^{4.4}) \cdot (1 + 18 \cdot f^{8})} - 0.16 \cdot g$$

$$\frac{\partial h}{\partial t} = 0.062 \cdot \frac{16 \cdot Red^{9.9}}{(1 + 16 \cdot Red^{9.9}) \cdot (1 + 5.9 \cdot g^{7.8})} - 0.33 \cdot h$$

$$\frac{\partial i}{\partial t} = 0.025 \cdot \frac{85 \cdot d^{9}}{(1 + 5.2 \cdot b^{3.1}) \cdot (1 + 85 \cdot d^{9})} - 0.32 \cdot i$$

$$\frac{\partial j}{\partial t} = 0.074 \cdot \frac{1}{(1 + 14 \cdot Green^{4})} - 0.56 \cdot j$$

$$\frac{\partial k}{\partial t} = 0.078 \cdot \frac{1}{(1 + 9 \cdot e^{2.9})} - 0.37 \cdot k$$

$$\frac{\partial l}{\partial t} = 0.075 \cdot \frac{1.7 \cdot Red^{7}}{(1 + 1.7 \cdot Red^{7})} - 0.43 \cdot l$$

Logo pattern (Fig. 8B):

$$\frac{\partial Blue}{\partial t} = 0.1 \cdot \frac{(58 \cdot f^{8.8} \cdot 9.7 \cdot h^{10} + 58 \cdot f^{8.8} + 9.7 \cdot h^{10}) \cdot 79 \cdot a^{6.8}}{(1 + 58 \cdot f^{8.8}) \cdot (1 + 9.7 \cdot h^{10}) \cdot (1 + 79 \cdot a^{6.8}) \cdot (1 + 97 \cdot b^{9.1})} - 0.1 \cdot Blue$$

$$\frac{\partial a}{\partial t} = 0.083 \cdot \frac{16 \cdot d^{6.4}}{(1 + 3.6 \cdot Red^{6.7}) \cdot (1 + 3.6 \cdot Green^{8.1}) \cdot (1 + 47 \cdot c^{2.3}) \cdot (1 + 16 \cdot d^{6.4})} - 0.18 \cdot a$$

$$\frac{\partial b}{\partial t} = 0.019 \cdot \frac{11 \cdot Red^{9.5} \cdot 10 \cdot Green^{9.9} \cdot 13 \cdot a^{10}}{(1 + 11 \cdot Red^{9.5}) \cdot (1 + 10 \cdot Green^{9.9}) \cdot (1 + 13 \cdot a^{10})} - 0.84 \cdot b$$

$$\frac{\partial c}{\partial t} = 0.004 \cdot \frac{6.8 \cdot Green^{6.1}}{(1 + 6.8 \cdot Green^{6.1}) \cdot (1 + 1.7 \cdot Red^{6.1})} - 0.59 \cdot c$$

$$\frac{\partial d}{\partial t} = 0.022 \cdot \frac{\left((38 \cdot Green^{2.8} \cdot 14 \cdot f^{7.7} + 38 \cdot Green^{2.8} + 14 \cdot f^{7.7}) \cdot 39 \cdot g^{7.5} + (38 \cdot Green^{2.8} \cdot 14 \cdot f^{7.7} + 38 \cdot Green^{2.8} + 14 \cdot f^{7.7}) + 39 \cdot g^{7.5}\right) \cdot 36 \cdot Red^{1.4}}{(1 + 38 \cdot Green^{2.8}) \cdot (1 + 14 \cdot f^{7.7}) \cdot (1 + 39 \cdot g^{7.5}) \cdot (1 + 36 \cdot Red^{1.4}) \cdot (1 + 97 \cdot c^{3})} - 0.29 \cdot d$$

$$\frac{\partial e}{\partial t} = 0.03 \cdot \frac{22 \cdot Red^{1.9} \cdot 24 \cdot Green^{4.5}}{(1 + 22 \cdot Red^{1.9}) \cdot (1 + 24 \cdot Green^{4.5})} - 0.94 \cdot e$$

$$\frac{\partial f}{\partial t} = 0.03 \cdot \frac{1}{(1 + 3.6 \cdot Red^{6.7}) \cdot (1 + 92 \cdot e^{9.1})} - 0.84 \cdot f$$

$$\frac{\partial g}{\partial t} = 0.091 \cdot \frac{10 \cdot Green^{9.9}}{(1 + 10 \cdot Green^{9.9})} - 0.22 \cdot g$$

$$\frac{\partial h}{\partial t} = 0.076 \cdot \frac{\left((11 \cdot Red^{10} \cdot 9.6 \cdot Green^{9.9} + 11 \cdot Red^{10} + 9.6 \cdot Green^{9.9}) \cdot 39 \cdot f^{10} + (11 \cdot Red^{10} \cdot 9.6 \cdot Green^{9.9} + 11 \cdot Red^{10} + 9.6 \cdot Green^{9.9}) + 39 \cdot f^{10}\right)}{(1 + 11 \cdot Red^{10}) \cdot (1 + 9.6 \cdot Green^{9.9}) \cdot (1 + 39 \cdot f^{10})} - 0.4 \cdot h$$

Letter 'U' pattern (Fig. 8B):

$$\frac{\partial Blue}{\partial t} = 0.1 \cdot \frac{(49 \cdot e^{9.9} \cdot 72 \cdot g^{4.4} + 49 \cdot e^{9.9} + 72 \cdot g^{4.4})}{(1 + 49 \cdot e^{9.9}) \cdot (1 + 72 \cdot g^{4.4}) \cdot (1 + 1e+02 \cdot a^{9.8}) \cdot (1 + 80 \cdot c^{10})} - 0.1 \cdot Blue$$

$$\frac{\partial a}{\partial t} = 0.0027 \cdot \frac{\left(\begin{array}{l} \left(\begin{array}{l} \left(\begin{array}{l} \left((2.6 \cdot Red^{9.9} \cdot 57 \cdot a^{5.3} + 2.6 \cdot Red^{9.9} + 57 \cdot a^{5.3}) \cdot 95 \cdot b^{9.3} + (2.6 \cdot Red^{9.9} \cdot 57 \cdot a^{5.3} + 2.6 \cdot Red^{9.9} + 57 \cdot a^{5.3}) + 95 \cdot b^{9.3}\right) \cdot 93 \cdot c^{9.8} \\ +\left((2.6 \cdot Red^{9.9} \cdot 57 \cdot a^{5.3} + 2.6 \cdot Red^{9.9} + 57 \cdot a^{5.3}) \cdot 95 \cdot b^{9.3} + (2.6 \cdot Red^{9.9} \cdot 57 \cdot a^{5.3} + 2.6 \cdot Red^{9.9} + 57 \cdot a^{5.3}) + 95 \cdot b^{9.3}\right) + 93 \cdot c^{9.8} \end{array} \right) \cdot 5.2 \cdot e^{6.3} + \\ \left(\begin{array}{l} \left((2.6 \cdot Red^{9.9} \cdot 57 \cdot a^{5.3} + 2.6 \cdot Red^{9.9} + 57 \cdot a^{5.3}) \cdot 95 \cdot b^{9.3} + (2.6 \cdot Red^{9.9} \cdot 57 \cdot a^{5.3} + 2.6 \cdot Red^{9.9} + 57 \cdot a^{5.3}) + 95 \cdot b^{9.3}\right) \cdot 93 \cdot c^{9.8} \\ +\left((2.6 \cdot Red^{9.9} \cdot 57 \cdot a^{5.3} + 2.6 \cdot Red^{9.9} + 57 \cdot a^{5.3}) \cdot 95 \cdot b^{9.3} + (2.6 \cdot Red^{9.9} \cdot 57 \cdot a^{5.3} + 2.6 \cdot Red^{9.9} + 57 \cdot a^{5.3}) + 95 \cdot b^{9.3}\right) + 93 \cdot c^{9.8} \end{array} \right) + 5.2 \cdot e^{6.3} \end{array} \right) \cdot 95 \cdot f^{10} \\ + \left(\begin{array}{l} \left(\begin{array}{l} \left((2.6 \cdot Red^{9.9} \cdot 57 \cdot a^{5.3} + 2.6 \cdot Red^{9.9} + 57 \cdot a^{5.3}) \cdot 95 \cdot b^{9.3} + (2.6 \cdot Red^{9.9} \cdot 57 \cdot a^{5.3} + 2.6 \cdot Red^{9.9} + 57 \cdot a^{5.3}) + 95 \cdot b^{9.3}\right) \cdot 93 \cdot c^{9.8} \\ +\left((2.6 \cdot Red^{9.9} \cdot 57 \cdot a^{5.3} + 2.6 \cdot Red^{9.9} + 57 \cdot a^{5.3}) \cdot 95 \cdot b^{9.3} + (2.6 \cdot Red^{9.9} \cdot 57 \cdot a^{5.3} + 2.6 \cdot Red^{9.9} + 57 \cdot a^{5.3}) + 95 \cdot b^{9.3}\right) + 93 \cdot c^{9.8} \end{array} \right) \cdot 5.2 \cdot e^{6.3} \\ + \left(\begin{array}{l} \left(\begin{array}{c} (2.6 \cdot Red^{9.9} \cdot 57 \cdot a^{5.3} + 2.6 \cdot Red^{9.9} + 57 \cdot a^{5.3}) \cdot 95 \cdot b^{9.3} + (2.6 \cdot Red^{9.9} \cdot 57 \cdot a^{5.3} + 2.6 \cdot Red^{9.9} + 57 \cdot a^{5.3}) + \\ 95 \cdot b^{9.3} \end{array} \right) \cdot 93 \cdot c^{9.8} \\ +\left((2.6 \cdot Red^{9.9} \cdot 57 \cdot a^{5.3} + 2.6 \cdot Red^{9.9} + 57 \cdot a^{5.3}) \cdot 95 \cdot b^{9.3} + (2.6 \cdot Red^{9.9} \cdot 57 \cdot a^{5.3} + 2.6 \cdot Red^{9.9} + 57 \cdot a^{5.3}) + 95 \cdot b^{9.3}\right) + 93 \cdot c^{9.8} \end{array} \right) + 5.2 \cdot e^{6.3} \end{array} \right) + 95 \cdot f^{10} \end{array} \right)}{(1 + 2.6 \cdot Red^{9.9}) \cdot (1 + 57 \cdot a^{5.3}) \cdot (1 + 95 \cdot b^{9.3}) \cdot (1 + 93 \cdot c^{9.8}) \cdot (1 + 5.2 \cdot e^{6.3}) \cdot (1 + 95 \cdot f^{10})} - 0.15 \cdot a$$

$$\frac{\partial b}{\partial t} = 0.096 \cdot \frac{1}{(1 + 3.6 \cdot Red^{5.3}) \cdot (1 + 53 \cdot Green^{6.4}) \cdot (1 + 96 \cdot c^{1.3})} - 0.22 \cdot b$$

$$\frac{\partial c}{\partial t} = 0.078 \cdot \frac{(20 \cdot Red^{5} \cdot 30 \cdot c^{3.6} + 20 \cdot Red^{5} + 30 \cdot c^{3.6}) \cdot 17 \cdot Green^{7.2}}{(1 + 20 \cdot Red^{5}) \cdot (1 + 30 \cdot c^{3.6}) \cdot (1 + 17 \cdot Green^{7.2}) \cdot (1 + 77 \cdot d^{1.6})} - 0.78 \cdot c$$

$$\frac{\partial d}{\partial t} = 0.084 \cdot \frac{\left((2.7 \cdot Red^{5.4} \cdot 5.2 \cdot Green^{6.8} + 2.7 \cdot Red^{5.4} + 5.2 \cdot Green^{6.8}) \cdot 9.8 \cdot d^{5.6} + (2.7 \cdot Red^{5.4} \cdot 5.2 \cdot Green^{6.8} + 2.7 \cdot Red^{5.4} + 5.2 \cdot Green^{6.8}) + 9.8 \cdot d^{5.6}\right)}{(1 + 2.7 \cdot Red^{5.4}) \cdot (1 + 5.2 \cdot Green^{6.8}) \cdot (1 + 9.8 \cdot d^{5.6}) \cdot (1 + 43 \cdot c^{1.9})} - 0.85 \cdot d$$

$$\frac{\partial e}{\partial t} = 0.033 \cdot \frac{(29 \cdot Red^{4.4} \cdot 94 \cdot c^{1.7} + 29 \cdot Red^{4.4} + 94 \cdot c^{1.7})}{(1 + 29 \cdot Red^{4.4}) \cdot (1 + 94 \cdot c^{1.7}) \cdot (1 + 4.8 \cdot Green^{3.4}) \cdot (1 + 85 \cdot f^{1.6}) \cdot (1 + 37 \cdot h^{8.6})} - 0.27 \cdot e$$

$$\frac{\partial f}{\partial t} = 0.091 \cdot \frac{1}{(1 + 70 \cdot Red^{2.7}) \cdot (1 + 19 \cdot Green^{5.7}) \cdot (1 + 97 \cdot c^{1.3})} - 0.49 \cdot f$$

$$\frac{\partial g}{\partial t} = 0.096 \cdot \frac{89 \cdot a^{2.7} \cdot 72 \cdot e^{9.3}}{(1 + 89 \cdot a^{2.7}) \cdot (1 + 72 \cdot e^{9.3})} - 0.1 \cdot g$$

$$\frac{\partial h}{\partial t} = 0.01 \cdot \frac{1}{(1 + 19 \cdot c^{1})} - 0.57 \cdot h$$

Letter 'M' pattern (Fig. 8B):

$$\frac{\partial Blue}{\partial t} = 0.098 \cdot \frac{(48 \cdot a^{10} \cdot 50 \cdot f^{9.9} + 48 \cdot a^{10} + 50 \cdot f^{9.9}) \cdot 43 \cdot h^{9.3}}{(1 + 48 \cdot a^{10}) \cdot (1 + 50 \cdot f^{9.9}) \cdot (1 + 11 \cdot c^{10}) \cdot (1 + 43 \cdot h^{9.3}) \cdot (1 + 11 \cdot i^{6.3})} - 0.11 \cdot Blue$$

$$\frac{\partial a}{\partial t} = 0.039 \cdot \frac{(61 \cdot b^{4.6} \cdot 85 \cdot g^{9.8} + 61 \cdot b^{4.6} + 85 \cdot g^{9.8})}{(1 + 61 \cdot b^{4.6}) \cdot (1 + 85 \cdot g^{9.8}) \cdot (1 + 4.3 \cdot Red^{1.1}) \cdot (1 + 1.7 \cdot Green^{7.8}) \cdot (1 + 3.8 \cdot e^{2.2}) \cdot (1 + 1.7 \cdot f^{5.7})} - 0.69 \cdot a$$

$$\frac{\partial b}{\partial t} = 0.044 \cdot \frac{86 \cdot Red^{5.4}}{(1 + 86 \cdot Red^{5.4}) \cdot (1 + 3.8 \cdot Green^{2.1}) \cdot (1 + 12 \cdot b^{10}) \cdot (1 + 83 \cdot d^{1.9})} - 0.99 \cdot b$$

$$\frac{\partial c}{\partial t} = 0.091 \cdot \frac{(38 \cdot a^{8.8} \cdot 98 \cdot d^{1} + 38 \cdot a^{8.8} + 98 \cdot d^{1}) \cdot 49 \cdot e^{9.9}}{(1 + 38 \cdot a^{8.8}) \cdot (1 + 98 \cdot d^{1}) \cdot (1 + 4.7 \cdot Red^{1.3}) \cdot (1 + 49 \cdot e^{9.9})} - 0.66 \cdot c$$

$$\frac{\partial d}{\partial t} = 0.085 \cdot \frac{\left((43 \cdot Red^{2.3} \cdot 46 \cdot b^{6.3} + 43 \cdot Red^{2.3} + 46 \cdot b^{6.3}) \cdot 18 \cdot g^{6.1} + (43 \cdot Red^{2.3} \cdot 46 \cdot b^{6.3} + 43 \cdot Red^{2.3} + 46 \cdot b^{6.3}) + 18 \cdot g^{6.1}\right)}{(1 + 43 \cdot Red^{2.3}) \cdot (1 + 46 \cdot b^{6.3}) \cdot (1 + 18 \cdot g^{6.1}) \cdot (1 + 83 \cdot Green^{2.7}) \cdot (1 + 9.1 \cdot e^{9.8})} - 0.44 \cdot d$$

$$\frac{\partial e}{\partial t} = 0.096 \cdot \frac{18 \cdot Green^{6.1} \cdot 34 \cdot b^{10}}{(1 + 18 \cdot Green^{6.1}) \cdot (1 + 34 \cdot b^{10}) \cdot (1 + 3.8 \cdot c^{1.3}) \cdot (1 + 75 \cdot d^{2.5})} - 0.3 \cdot e$$

$$\frac{\partial f}{\partial t} = 0.056 \cdot \frac{(7.6 \cdot Green^{7.9} \cdot 79 \cdot d^{3.5} + 7.6 \cdot Green^{7.9} + 79 \cdot d^{3.5}) \cdot 62 \cdot c^{2} \cdot 98 \cdot e^{2}}{(1 + 7.6 \cdot Green^{7.9}) \cdot (1 + 79 \cdot d^{3.5}) \cdot (1 + 62 \cdot c^{2}) \cdot (1 + 98 \cdot e^{2})} - 0.16 \cdot f$$

$$\frac{\partial g}{\partial t} = 0.017 \cdot \frac{43 \cdot b^{8.8}}{(1 + 43 \cdot b^{8.8}) \cdot (1 + 4.3 \cdot e^{6.9})} - 0.27 \cdot g$$

$$\frac{\partial h}{\partial t} = 0.076 \cdot \frac{44 \cdot a^{9.9} \cdot 34 \cdot Red^{7.9}}{(1 + 44 \cdot a^{9.9}) \cdot (1 + 34 \cdot Red^{7.9}) \cdot (1 + 23 \cdot b^{8.8}) \cdot (1 + 9.8 \cdot g^{3.1})} - 0.34 \cdot h$$

$$\frac{\partial i}{\partial t} = 0.029 \cdot \frac{8.5 \cdot c^{5.3}}{(1 + 8.5 \cdot c^{5.3})} - 0.13 \cdot i$$

Letter 'B' pattern (Fig. 8B):

$$\frac{\partial Blue}{\partial t} = 0.1 \cdot \frac{\left((50 \cdot b^{9.6} \cdot 90 \cdot h^{9.5} + 50 \cdot b^{9.6} + 90 \cdot h^{9.5}) \cdot 50 \cdot i^{9.2} + (50 \cdot b^{9.6} \cdot 90 \cdot h^{9.5} + 50 \cdot b^{9.6} + 90 \cdot h^{9.5}) + 50 \cdot i^{9.2}\right)}{(1 + 50 \cdot b^{9.6}) \cdot (1 + 90 \cdot h^{9.5}) \cdot (1 + 50 \cdot i^{9.2}) \cdot (1 + 18 \cdot d^{10}) \cdot (1 + 15 \cdot e^{8.7})} - 0.1 \cdot Blue$$

$$\frac{\partial a}{\partial t} = 0.093 \cdot \frac{\left((1.5 \cdot Green^{5.4} \cdot 78 \cdot a^{3.5} + 1.5 \cdot Green^{5.4} + 78 \cdot a^{3.5}) \cdot 38 \cdot f^{9.8} + (1.5 \cdot Green^{5.4} \cdot 78 \cdot a^{3.5} + 1.5 \cdot Green^{5.4} + 78 \cdot a^{3.5}) + 38 \cdot f^{9.8}\right) \cdot 44 \cdot Red^{5.9}}{(1 + 1.5 \cdot Green^{5.4}) \cdot (1 + 78 \cdot a^{3.5}) \cdot (1 + 38 \cdot f^{9.8}) \cdot (1 + 44 \cdot Red^{5.9}) \cdot (1 + 86 \cdot c^{1.2})} - 0.53 \cdot a$$

$$\frac{\partial b}{\partial t} = 0.066 \cdot \frac{38 \cdot Red^{9.9} \cdot 19 \cdot Green^{2} \cdot 38 \cdot k^{8.2}}{(1 + 38 \cdot Red^{9.9}) \cdot (1 + 19 \cdot Green^{2}) \cdot (1 + 67 \cdot a^{1}) \cdot (1 + 96 \cdot c^{1.2}) \cdot (1 + 15 \cdot d^{9.2}) \cdot (1 + 11 \cdot g^{7.8}) \cdot (1 + 6.6 \cdot i^{1.1}) \cdot (1 + 38 \cdot k^{8.2})} - 0.72 \cdot b$$

$$\frac{\partial c}{\partial t} = 0.083 \cdot \frac{1.5 \cdot Red^{7.2}}{(1 + 1.5 \cdot Red^{7.2}) \cdot (1 + 16 \cdot c^{1.8}) \cdot (1 + 22 \cdot e^{9.5})} - 0.14 \cdot c$$

$$\frac{\partial d}{\partial t} = 0.083 \cdot \frac{15 \cdot Red^{7.2} \cdot 81 \cdot k^{7.3}}{(1 + 15 \cdot Red^{7.2}) \cdot (1 + 94 \cdot b^{1.7}) \cdot (1 + 38 \cdot c^{8.1}) \cdot (1 + 6.6 \cdot d^{3.1}) \cdot (1 + 81 \cdot k^{7.3})} - 0.43 \cdot d$$

$$\frac{\partial e}{\partial t} = 0.07 \cdot \frac{24 \cdot b^{6.9}}{(1 + 24 \cdot b^{6.9}) \cdot (1 + 6.6 \cdot Green^{6.1}) \cdot (1 + 1e + 02 \cdot d^{9.6}) \cdot (1 + 68 \cdot g^{9.2})} - 0.38 \cdot e$$

$$\frac{\partial f}{\partial t} = 0.025 \cdot \frac{1}{(1 + 85 \cdot Red^{1.2}) \cdot (1 + 20 \cdot Green^{6.7})} - 0.32 \cdot f$$

$$\frac{\partial g}{\partial t} = 0.045 \cdot \frac{1}{(1 + 44 \cdot Red^{8.5}) \cdot (1 + 11 \cdot g^{7.8})} - 0.3 \cdot g$$

$$\frac{\partial h}{\partial t} = 0.1 \cdot \frac{(38 \cdot b^{8.6} \cdot 66 \cdot e^{8.6} + 38 \cdot b^{8.6} + 66 \cdot e^{8.6})}{(1 + 38 \cdot b^{8.6}) \cdot (1 + 66 \cdot e^{8.6}) \cdot (1 + 11 \cdot Red^{7.8}) \cdot (1 + 6.6 \cdot i^{1.8})} - 0.5 \cdot h$$

$$\frac{\partial i}{\partial t} = 0.06 \cdot \frac{\left((76 \cdot f^{1.3} \cdot 82 \cdot h^{8} + 76 \cdot f^{1.3} + 82 \cdot h^{8}) \cdot 25 \cdot i^{9.8} + (76 \cdot f^{1.3} \cdot 82 \cdot h^{8} + 76 \cdot f^{1.3} + 82 \cdot h^{8}) + 25 \cdot i^{9.8}\right) \cdot 8.4 \cdot Red^{9.3} \cdot 41 \cdot j^{5.9}}{(1 + 76 \cdot f^{1.3}) \cdot (1 + 82 \cdot h^{8}) \cdot (1 + 25 \cdot i^{9.8}) \cdot (1 + 8.4 \cdot Red^{9.3}) \cdot (1 + 41 \cdot j^{5.9})} - 0.16 \cdot i$$

$$\frac{\partial j}{\partial t} = 0.042 \cdot \frac{90 \cdot Green^{9.5}}{(1 + 90 \cdot Green^{9.5}) \cdot (1 + 7.4 \cdot Red^{4.6}) \cdot (1 + 99 \cdot d^{1}) \cdot (1 + 8.8 \cdot e^{1.1}) \cdot (1 + 88 \cdot g^{1.1})} - 0.12 \cdot j$$

$$\frac{\partial k}{\partial t} = 0.05 \cdot \frac{1}{(1 + 8.1 \cdot f^{1.4})} - 0.44 \cdot k$$

Letter 'C' pattern (Fig. 8B):

$$\frac{\partial Blue}{\partial t} = 0.099 \cdot \frac{13 \cdot c^{9.8}}{(1 + 13 \cdot c^{9.8})} - 0.12 \cdot Blue$$

$$\frac{\partial a}{\partial t} = 0.0097 \cdot \frac{41 \cdot Red^{2.1} \cdot 45 \cdot Green^{9.4}}{(1 + 41 \cdot Red^{2.1}) \cdot (1 + 45 \cdot Green^{9.4}) \cdot (1 + 44 \cdot d^{3.5})} - 0.23 \cdot a$$

$$\frac{\partial b}{\partial t} = 0.093 \cdot \frac{24 \cdot Red^{3.7} \cdot 40 \cdot Green^{5.2} \cdot 61 \cdot e^{2.8}}{(1 + 24 \cdot Red^{3.7}) \cdot (1 + 40 \cdot Green^{5.2}) \cdot (1 + 76 \cdot d^{5.4}) \cdot (1 + 61 \cdot e^{2.8})} - 0.52 \cdot b$$

$$\frac{\partial c}{\partial t} = 0.093 \cdot \frac{48 \cdot a^{9.3}}{(1 + 3.2 \cdot Red^{3.5}) \cdot (1 + 48 \cdot a^{9.3}) \cdot (1 + 22 \cdot b^{9.4}) \cdot (1 + 60 \cdot d^{9.5})} - 0.27 \cdot c$$

$$\frac{\partial d}{\partial t} = 0.015 \cdot \frac{8.4 \cdot Green^{1.4}}{(1 + 8.4 \cdot Green^{1.4}) \cdot (1 + 3.2 \cdot b^{1.6})} - 0.69 \cdot d$$

$$\frac{\partial e}{\partial t} = 0.079 \cdot \frac{40 \cdot Green^{5.8}}{(1 + 40 \cdot Green^{5.8}) \cdot (1 + 7.6 \cdot Red^{2.5})} - 0.63 \cdot e$$

Gap gene expression pattern (Fig. 9):

$$\frac{\partial Hb}{\partial t} = 0.099 \cdot \frac{17 \cdot Bcd^{3.5}}{(1 + 17 \cdot Bcd^{3.5}) \cdot (1 + 100 \cdot Kni^{1.8})} - 0.11 \cdot Hb$$

$$\frac{\partial Kr}{\partial t} = 0.093 \cdot \frac{4 \cdot Bcd^{9.9} \cdot 4.1 \cdot Cad^{7.6}}{(1 + 4 \cdot Bcd^{9.9}) \cdot (1 + 4.1 \cdot Cad^{7.6}) \cdot (1 + 1.1 \cdot Gt^{4.8})} - 0.1 \cdot Kr$$

$$\frac{\partial Kni}{\partial t} = 0.1 \cdot \frac{7.3 \cdot Bcd^{9.2} \cdot 1.9 \cdot Cad^{10}}{(1 + 7.3 \cdot Bcd^{9.2}) \cdot (1 + 1.9 \cdot Cad^{10}) \cdot (1 + 1.1 \cdot Hb^{9.5}) \cdot (1 + 1.1 \cdot Kr^{6.6}) \cdot (1 + 1.1 \cdot Gt^{9.8})} - 0.1 \cdot Kni$$

$$\frac{\partial Gt}{\partial t} = 0.087 \cdot \frac{11 \cdot Bcd^{5.1}}{(1 + 11 \cdot Bcd^{5.1}) \cdot (1 + 99 \cdot Kr^{5.7})} - 0.11 \cdot Gt$$
